# Editorial: Angiogenesis and Nutraceuticals

**DOI:** 10.3389/fphar.2022.943158

**Published:** 2022-05-30

**Authors:** Lorenza Trabalzini, Rosa Tundis, Giuseppe Valacchi, Pablo Andres Evelson, Federica Finetti

**Affiliations:** ^1^ Department of Biotechnology, Chemistry and Pharmacy, University of Siena, Siena, Italy; ^2^ Department of Pharmacy, Health and Nutritional Sciences, University of Calabria, Rende, Italy; ^3^ Plants for Human Health Institute, North Carolina State University, Kannapolis, NC, United States; ^4^ Faculty of Pharmacy and Biochemistry, University of Buenos Aires, Buenos Aires, Argentina

**Keywords:** angiogenesis, nutraceuticals, endothelial cells, foods, food-derived products

Angiogenesis is the formation of new capillary blood vessels from pre-existing arteries, veins and capillaries. This process involves proliferation, migration and differentiation of vascular endothelial cells following the stimulation by specific angiogenic factors. Angiogenesis is a hallmark of several physiological and pathological conditions. It contributes to tissue repair, expansion, and remodeling in physiological processes such as wound healing, ovulation and embryo development. Nevertheless, when dysregulated, angiogenesis contributes to disease progression in different pathologies including cancer, atherosclerosis, cerebrovascular diseases and chronic inflammation. Many of these conditions share the same characteristics, including the occurrence of hypoxia, inflammation or oxidative stress, recruitment of inflammatory cells, angiogenic growth factor production, basement membrane degradation, endothelial cell migration, proliferation and differentiation and modulation of vascular support cells.

Targeting the angiogenic process is at the present one of the main goals in pharmacology and there has been a great interest in the development of angiogenesis strategies that could inhibit tumor vascularization, diabetic retinopathy, inflammatory or cerebrovascular diseases. At present, the antiangiogenic therapy utilizes drugs that target either the angiogenic factors or angiogenic factor signal cascade. However, several clinical trials have shown that these angiogenic regulators have clinical limitations including short therapeutic effects, development of resistance and adverse reactions which have led to the decrease in the use of anti-angiogenic drugs and have encouraged the search for alternative strategies to treat angiogenic diseases.

The term nutraceuticals refers to dietary components that provide health benefit in addition to their basic nutritional value. In the last years, the diet-based approach for the treatment of angiogenesis is gaining more attention, as testified by several reports showing the anti-angiogenesis effect of food components. A wide variety of bioactive compounds identified specifically in plants are known to possess anti-angiogenic effects by modulating different pathways. Thus, identification of natural food components for preventing or ameliorating angiogenesis-associated complications would be of a greater advantage because of their low toxicity or limited side effects.

A comprehensive review of the role of chemical components of foods in the regulation of angiogenesis is provided by Pan et al.. Pharmacological properties of flavonoids, terpenoids, saponins, alkaloids, polysaccharides, tannins are described, with a particular focus on their ability to inhibit or promote angiogenesis in different models. These bioactive compounds can regulate the transmission of different angiogenesis-related signaling pathways. In particular, the inhibition of growth and migration of vascular endothelial cells, as well as the regulation of the angiogenic factors is one of the basic anti-angiogenic mechanisms of food active components. The use of functional foods or bioactive food components as regulators of angiogenesis is proposed not only to treat tumors and cardiovascular diseases, but also for the prevention and treatment of other angiogenesis-related diseases, including skin wounds and ocular conditions related to aberrant vessels formation.

Among functional foods, there is a global consensus about the protective role of fruits and vegetables, while there is no clear evidence about the effects of fruit juices consuming, due to their lower fiber content and higher caloric density in comparison with the fresh fruits (Smeriglio et al.). However, it has been shown that fruit juices without additives as *Citrus* juice retain the majority of bio-active compounds of the parent fruit. Smeriglio et al. have investigated the chemical profile and the biological properties of the juice of *Citrus lumia* Risso, an ancient Mediterranean *Citrus* fruit. This original study shows that *C. lumia* juice has antioxidant, anti-inflammatory and anti-angiogenic properties. These activities are due to the marked presence of phenolic acids, flavonoids and ascorbic acid. This study sheds light on the main biological activities of this plant-derived food, although its potential is limited by the low availability of *C.lumia* fruits. However, the promising results of this study may pave the way to an increase in crop production of this ancient species of *Citrus*.

The use of foods or food-derived products to prevent and cure diseases is part of traditional medicine and it dates back to ancient times. It is generally linked to an empirical use without very often knowing the detailed mechanism of action.

Shexiang Baoxin Pill (SBP) is an oral formulation of traditional Chinese medicine used for the treatment of coronary heart diseases. It displays pleiotropic roles in protecting the cardiovascular system. However, the mode of action of SBP in promoting angiogenesis, and in particular the synergy between its constituents is currently not fully understood. Choi et al. focused on the combination of two SBP constituents derived from *Panax ginseng,* ginsenoside Rb2 and its hydrolytic product Rg3, in order to investigate its molecular mode of action associated with angiogenesis in endothelial cell models. In this study it has been provided the first evidence that ginsenoside Rb2 and ginsenoside Rg3 synergistically promote endothelial cell proliferation and protect them against homocysteine-induced damage. Rb2 and Rg3 combination promotes endothelial cell proliferation through the CXCR1/2 CXCL8 (IL8)-mediated PI3K/Akt and MAPK/ERK signaling pathway that may further affects angiogenesis. Overall, in this study the authors gained insight into the mode of action of a combination of two compounds from an originally more complex formulation, which could be beneficial to standardize and simplify its complexity while maintaining efficacy.

Compared with traditional therapy, the use of functional foods or food-derived products is safer and more easily accepted by patients. Nevertheless, the currently available knowledge on these treatments has many limitations (Pan et al.): 1) current research is limited to the use of cells and animal models, and there is no regulation of clinical trials, thus the translation of *in vitro* and *in vivo* results on the clinical setting still represents an obstacle to overcome; 2) most studies on the pharmacological activity of natural compounds only indicate a dose range and no indications are available regarding a precise dosage; 3) the regulatory mechanism of functional foods and their active components is not clear as in most cases they consist of mixtures of compounds.

In conclusion, the above works presented in this special Research Topic highlight the enormous potential of functional foods and bioactive constituents of diet in the prevention and treatment of angiogenesis-related diseases, even though a deeper understanding of their mechanisms of action and better designed clinical trials are needed.

